# Investigation of comorbidities of COVID-19 patients with hepatosteatosis using latent class analysis

**DOI:** 10.3389/fpubh.2022.990848

**Published:** 2022-09-29

**Authors:** Ozge Pasin, Sirin Cetin, Ahmet Turan Kaya

**Affiliations:** ^1^Department of Biostatistics, Faculty of Medicine, Bezmialem University, Istanbul, Turkey; ^2^Department of Biostatistics, Faculty of Medicine, Amasya University, Amasya, Turkey; ^3^Department of Radiology, Sabuncuoglu Serefeddin Research and Education Hospital, Amasya University, Amasya, Turkey

**Keywords:** liver, classification, statistics, multivariate analysis, latent

## Abstract

**Introduction:**

Coronavirus Disease 2019 (COVID-19) disease first appeared in Wuhan, China in December 2019. Subsequently, the pandemic spread rapidly throughout the entire world. The number of people who died from COVID-19 is rising daily due to the growing number cases. This retrospective study aims to classify patients with hepatosteatosis (HS) who had COVID-19, depending on additional disease characteristics and to compare survival times and death rates.

**Material and methods:**

The study included 433 individuals with COVID-19 and HS at Amasya University Sabuncuoglu Serefeddin Education and Research Hospital. Additional disease characteristics of patients with HS were analyzed using latent class analysis (LCA) and the patients were divided into two groups.

**Results:**

The study results indicate that the survival time of the first group, which was formed as a result of the LCA, was significantly lower than that of the second group (*p* = 0.038). The rate of diabetes, coronary artery disease, chronic rhythm disorder, chronic obstructive pulmonary disease (COPD) and chronic kidney disease was significantly higher in group 1 than in group 2 (respectively *p* < 0.001; *p* < 0.001; *p* < 0.001; *p* < 0.001; *p* = 0.015).

**Discussion:**

In patients with HS, the presence of diabetes, coronary artery disease, chronic rhythm problem, COPD, and chronic renal disorders contributes to an increase in death rates due to COVID-19.

## Introduction

Coronavirus Disease 2019 (COVID-19) first appeared in December 2019 Wuhan, China, and quickly became a worldwide pandemic with an increasing number of mortalities (1). In Fang et al.'s review of the comorbidities of patients with COVID-19, advanced age, male gender, chronic kidney disease, chronic obstructive pulmonary disease (COPD), and cardiovascular diseases were identified as significant factors exacerbating the condition. Moreover, evaluating the heart, liver, and kidneys when examining the area outside the lung during a routine chest computed tomography (CT) is essential. Hepatosteatosis (HS) worsens COVID-19 infections by increasing the inflammatory response along with the deterioration of liver functions (2–8). Furthermore, HS negatively affects the immune system and has been associated with recurrent infections and increased mortality (9–11). The prevalence of HS in the Turkish population is high (45.5%); therefore, it is a crucial issue in the country when associated with COVID-19. The presence of HS, along with possible additional diseases, is a significant comorbidity in COVID-19. Therefore, patients' survival times and other disease states should be evaluated. In addition to prolonged hospital stays, the presence of disease histories, such as hypertension, diabetes mellitus (DM), coronary artery disease (CAD), chronic heart disease (CHD), and chronic obstructive pulmonary disease (COPD), has significant effects on the survival time of patients with COVID-19.

This study used latent class analysis (LCA), an advanced statistical method, for the classification, and this increases the originality of the study. LCA often identifies qualitatively different subgroups within populations with specific characteristics. Using this method, the subgroups obtained from the analysis are called latent classes (12, 13). LCA classifies individuals into clusters based on the probability of membership estimated directly from the model. In LCA, clusters are revealed by interrelated observations through categorical variables (13, 14). LCA can be considered as an alternative to factor analysis for categorical variables. The primary advantage of LCA is that it is probabilistic and the model fit is based on measures. Therefore, it is less dependent on the researcher's reasoning than traditional approaches. In the literature, the effect of different diseases on the prognosis of patients with COVID-19 according to the presence of HS has not been evaluated using LCA (15).

The aim of this study is to classify patients with HS who have had COVID-19 depending on additional disease characteristics and to compare survival times and death rates of classes using LCA.

## Materials and methods

This retrospective and single-center study was approved by the Ethical Committee of Amasya University Sabuncuoglu Serefeddin Education and Research Hospital and was conducted according to the Declaration of Helsinki and Good Clinical Practice (10 December 2021, No: 2021/46711).

Ethical Considerations Protocol was approved by research and ethic committees and the institutional review board; all participants signed an informed consent.

### Study population and data collection

The data of 433 patients with COVID-19 who were admitted to the emergency department of Amasya University Sabuncuoglu Serefeddin Education and Research Hospital between July 2021 and November 2021 were collected. All patients who met the study's inclusion criteria during the time period were included. It is known that at least 20 times the number of variables is sufficient in multivariate analyses. In our study, based on LCA, at least 160 individuals should be recruited, as there were eight variables. When the proportion of subjects in group one is taken 0.50, the expected hazard ratio (HR) was taken 0.70, a minimum 247 patients should be included in the study with 95% reliability, 80% power. The inclusion criteria for the study were adult patients (age ≥ 18 years), patients with a positive Reverse Transcription Polymerase Chain Reaction (RT-PCR) test, and a chest CT, including the liver and spleen. Patients with a negative RT-PCR test, those given contrast material, those with image artifacts that hindered evaluation, those with chronic liver disease, those with solid or cystic lesions in the liver parenchyma, and pregnant women were excluded from the study. Patient's demographic information, comorbidities, hospital or intensive care unit (ICU) admission history, laboratory findings, length of hospital stay, and survival were recorded from electronic medical records. Laboratory examinations one day from the date corresponding to the first chest CT scan at the time of admission to the hospital were also recorded from electronic medical records. Patients' demographic information, comorbidities, hospital or intensive care unit admission history, laboratory findings, length of hospital stay, and survival were recorded from electronic medical records. Laboratory examinations within one day from the date corresponding to the first chest CT scan at the time of admission to the hospital were also recorded from electronic medical records.

**Figure 1:**
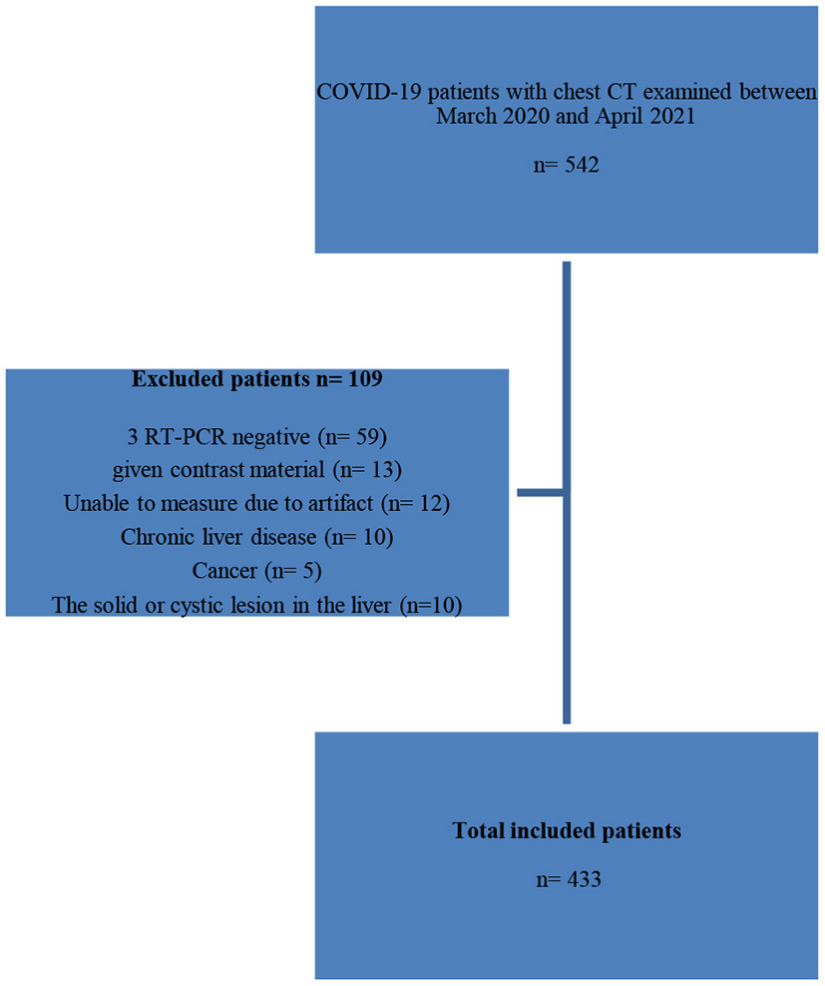
Flow cart of patients.

The selection of the study patients is shown using a study flow diagram (Figure 1).

### Clinical and laboratory data

The laboratory results obtained within one day from the initial chest CT date and comorbidities such as diabetes, hypertension, chronic renal failure, chronic lung and cardiovascular diseases, admission to the hospital and/or intensive care unit (ICU), and dates of death were scanned from the hospital's electronic medical records. The patient's length of stay in the hospital and their survival were recorded.

### CT protocol

The non-contrast chest CT scans were performed using multidetector CT (MDCT) 128-slice GE Healthcare Revolution EVO CT scanners (GE Medical Systems, Milwaukee, WI, USA). The settings were tube voltage, 120 kV; tube current, 100 mA−450 mA; beam collimation, 64 mm × 0.625 mm; beam pitch, 1.375; gantry rotation, 0.4 s; acquisition direction, craniocaudal; reconstruction kernel, standard; slice thickness, 0.625 mm; and section overlap, 0.625 mm. All chest CT scans were assessed at a lung window of 1,500 WW and −450 WL and a mediastinal window of 400 WW and 40 WL. The non-contrast chest CT was acquired during a single breath-hold. Craniocaudal axial images were obtained from the beginning of the thorax to the abdomen (the middle part of the kidneys) with the patient in a deep inspiration breath-hold and in a supine position.

### Image analysis

The first chest CT scan taken on admission to the hospital was evaluated for the presence of COVID-19 pneumonia and HS based on suspected COVID-19 lung involvement (1 = very low, 2 = low, 3 = uncertain, 4 = high, and 5 = very high were typical findings). The COVID-19 Reporting and Data System (CO-RADS) scores were assigned (16). In the qualitative analysis, the patient's liver density in the upper abdominal sections was included in the image, separated by the hepatic veins. This was measured by averaging the Hounsfield unit (HU) of a circular area of regions of interest (ROIs) from four different areas of both lobes. Spleen density was obtained from an ROI in the range of 1.4 cm^2^−1.8 cm^2^ placed in the parenchyma. In both organs, ROIs were placed in parenchyma areas at least 1 cm from low-density vascular structures, hilum and high-density (e.g., calcification) areas. HS was defined if the hepatic-to-splenic attenuation ratio (CTL/S) of these mean densities was less than 1 (17–19). Additional disease characteristics of patients with fatty liver were examined using LCA, and the patients were divided into two groups. Survival times, comorbidity rates and mortality rates were compared among the groups formed as a result of LCA.

### Statistical analysis

Descriptive statistics of categorical variables in the data set are given as numbers and percentages, and descriptive statistics for numerical variables are given as mean, standard deviation, median, minimum and maximum. The most appropriate class number was determined by comparing the latent class analysis and AIC (Akaike information criterion) and BIC (Bayesian information criterion) by evaluating the additional disease conditions of the patients. As a result of latent class analysis, two groups were obtained and the patients were divided into appropriate classes. The graphs of the patients' survival times were analyzed with Kaplan Meier curves. The logrank test was used to determine whether there was a significant difference in survival time between the classes. Relationships between categorical variables were analyzed with Pearson chi-square and Fisher exact tests. The statistical significance level was taken as 0.05. Statistical analyses were performed by using IBM SPSS Statistics for Windows, Version 27.0 (IBM. Armonk, NY) and R package.

## Results

The study results indicate that the mean age of 433 patients with HS and COVID-19 was 64.73 ± 13.988 years. Additionally, 60% (260) of the patients were female and 40% (173) were male. Furthermore, 66.1% (286) of the patients died due to COVID-19, 34.6% (150) died due to DM, 18.9% (82) died due to CAD, 7.6% (33) died due to chronic heart rhythm disorder, 1.4% (6) died due to peripheral artery diseases (PAD), 10.6% (46) died due to asthma, 6.9% (20) died due to COPD, 4.2% (18) died due to venous insufficiency, and 2.1% (9) died due to CKD (Table 1).

**Table 1:** Descriptive statistics of demographic features and comorbities of patients.

		**Frequency**	**Percent**
Gender	Female	260	60.0
	Male	173	40.0
Status	Death	286	66.1
	Alive	147	33.9
Diabetes mellitus (DM)	No	283	65.4
	Yes	150	34.6
Coronary artery disease (CAD)	No	351	81.1
	Yes	82	18.9
Chronic heart diseases (CHD)	No	400	92.4
	Yes	33	7.6
Peripheral artery disease (PAD)	No	427	98.6
	Yes	6	1.4
Asthma	No	387	89.4
	Yes	46	10.6
Chronic obstructive pulmonary diseases (COPD)	No	403	93.1
	Yes	30	6.9
Venousin sufficiency	No	415	95.8
	Yes	18	4.2
Chronic kidney disease (CKD)	No	424	97.9
	Yes	9	2.1

**Figure 2:**
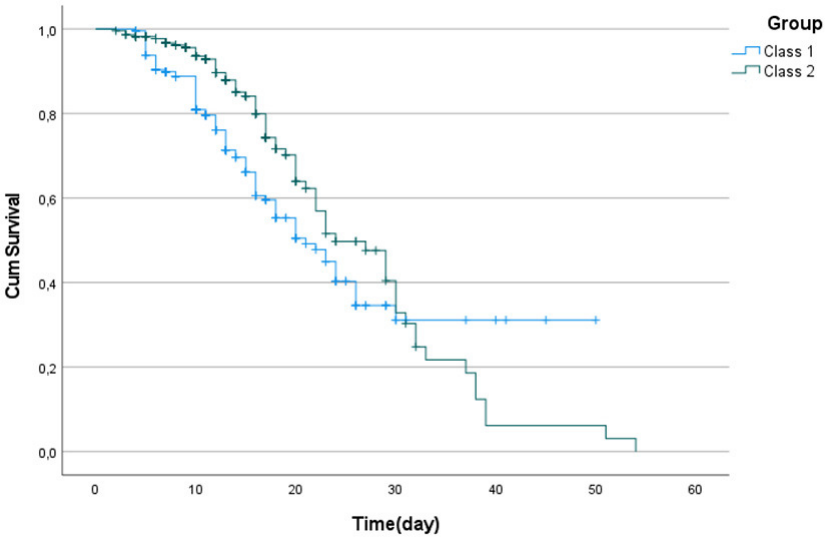
Kaplan Meier curve for patients by classes.

When we performed the LCA according to the comorbidities of the patients, the AIC value obtained from the model was 2038 and the BIC value was 2107. According to the LCA, the probability of an individual being in the first group was 0.536, and the probability of an individual being in the second group was 0.464. The results of the analysis indicated a substantial difference in length of hospital stay between the two groups generated by applying the comorbidities in the table above. The patients were divided into two groups according to their comorbidities. Patients in group 1 are those who are most at risk, which typically includes those who have DM, CAD, chronic rhythm disorders, COPD and CKD comorbidities. Group 2, the group with lower risk of death, mostly includes patients with CKD comorbidities. The survival time of the first group was significantly lower than that of the second group (p = 0.038). It was found that the median survival time of the first group was 21 and the median survival time of the second group was 24. The median times and 95% confidence intervals (CI) of the groups are presented in Table 2. Figure 2 shows the Kaplan–Meier graph for the two groups. The cumulative proportion surviving can be seen in Table 3 (Table 2, Figure 2).

**Table 2:** Median survival times of latent classes.

**Classes**	**Median**			
	**Estimate**	**Standard error**	**95% confidence interval**	
			**Lower bound**	**Upper bound**
Class 1	21.000	2.280	16.532	25.468
Class 2	24.000	2.531	19.040	28.960
Overall	23.000	1.084	20.876	25.124

**Table 3:** Cumulative proportion surviving at the time intervals for classes.

**Class**	**Time**	**Status**	**Cumulative proportion surviving**		**N of cumulative**
			**at the time**		**events**
			**Estimate**	**Std. error**	
Class 1	4	1	0.995	0.005	1
	10	1	0.809	0.028	38
	20	0	0.505	0.046	71
	30	0	0.311	0.058	82
	40	0	0.311	0.058	82
	50	0	0.311	0.058	82
Class 2	4	1	0.982	0.009	4
	10	1	0.936	0.018	12
	20	1	0.639	0.051	39
	30	1	0.329	0.065	54
	40	1	0.062	0.041	63
	51	1	0.031	0.030	64

**Figure 3:**
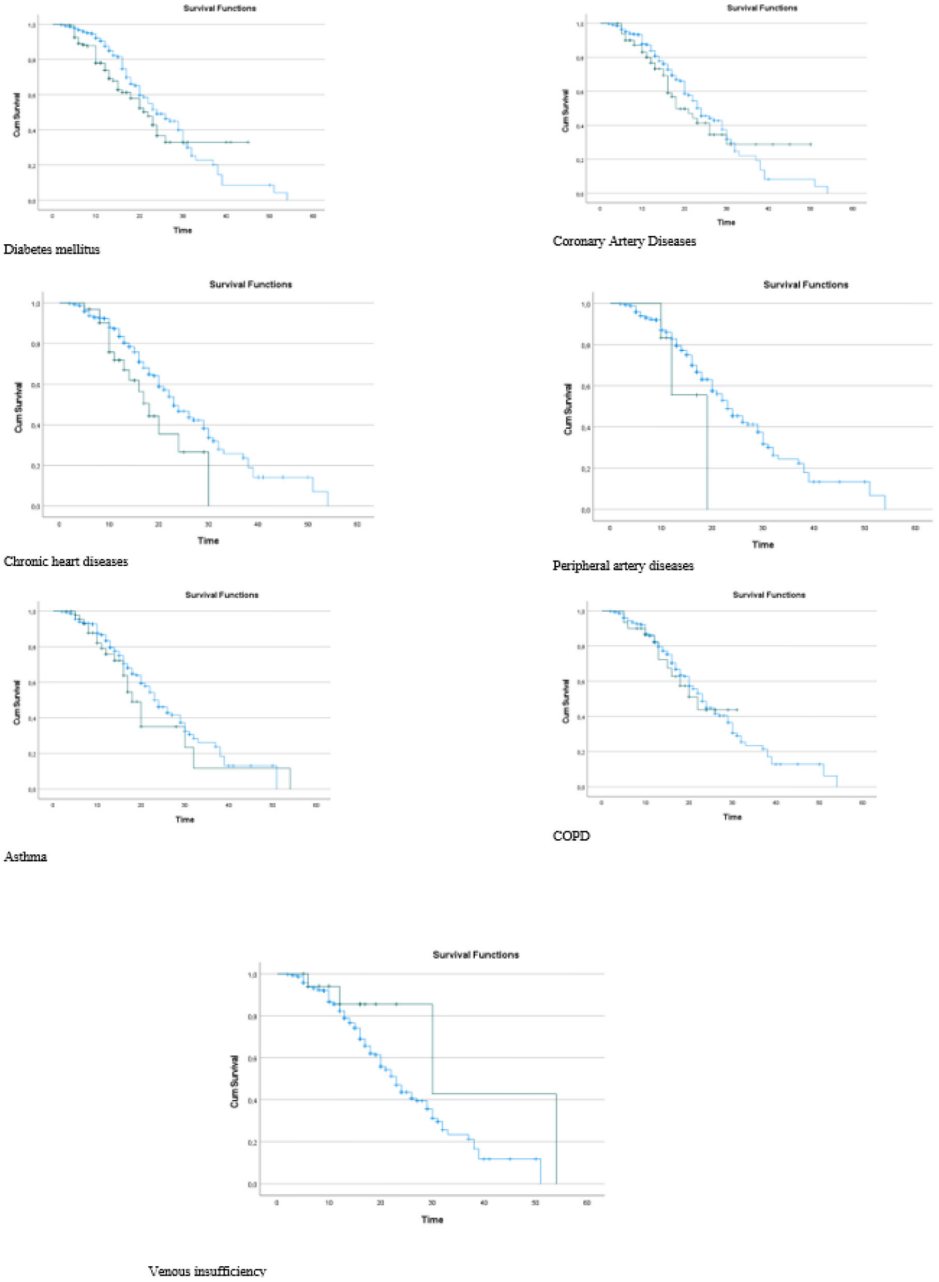
Kaplan Meier curves stratified by comorbidities.

It was observed that the death rates of the individuals in group 1 were significantly higher than those in group 2 when the death rates due to COVID-19 were examined according to the groups generated as a result of the LCA (*p* = 0.035). In other words, we can see that group 1 includes patients at higher risk (Table 4). It was found that the risk in group 1 was 1.403 times higher than that in group 2, and the HR was statistically significant (*p* = 0.044; HR: 1.403; CI of HR: 1.010–1.951). In Figure 3, Kaplan–Meier curves stratified by comorbidities can be seen (Figure 3).

**Table 4:** Survival status distributions of latent classes.

			**Class**		***p***
			**Class 1**	**Class 2**	
Status	Alive	n	129	157	0.035
		%	61.1	70.7	
	Death	n	82	65	
		%	38.9	29.3	
Total		n	211	222	433
		%	100	100	100

Comparisons were made with the groups generated as a result of the analysis in terms of comorbidities. The p values and distributions of the comparisons are provided in Table 5. As shown in Table 5, no significant difference was observed between group 1 and group 2 in terms of CAD, asthma, and venous insufficiency distributions (*p* = 0.439, *p* = 0.621, and *p* = 0.283, respectively). Diseases that were effective in separating the two groups were determined as DM, CAD, CHD, COPD, and CKD. The rates of DM, CAD, chronic rhythm disorder, COPD, and CKD were significantly higher in group 1 than in group 2 (*p* < 0.001, *p* < 0.001, *p* < 0.001, *p* < 0.001, and *p* = 0.015 respectively). Therefore, group 1, the group with a high risk of death, mostly includes patients with DM, CAD, chronic rhythm disorders, and COPD. Group 2, the group with a lower risk of death, mostly includes patients with CKD comorbidities (Table 5).

**Table 5:** Relations between classes and comorbidities.

			**Class**		***p***
			**Class 1**	**Class 2**	
Diabetes mellitus (DM)	No	n	63	220	<0.001
		%	29.9	99.1	
	Yes	n	148	2	
		%	70.1	0.9	
Coronary artery diseases (CAD)	No	n	130	221	<0.001
		%	61.6	99.5	
	Yes	n	81	1	
		%	38.4	0.5	
Chronic heart diseases (CHD)	No	n	178	222	<0.001
		%	84.4	100	
	Yes	n	33	0	
		%	15.6	0	
Peripheral artery diseases (PAD)	No	n	207	220	0.439
		%	98.1	99.1	
	Yes	n	4	2	
		%	1.9	0.9	
Asthma	No	n	187	200	0.621
		%	88.6	90.1	
	Yes	n	24	22	
		%	11.4	9.9	
Chronic obstructive pulmonary	No	n	182	221	<0.001
diseases (COPD)		%	86.3	99.5	
	Yes	n	29	1	
		%	13.7	0.5	
Venous insufficiency	No	n	200	215	0.283
		%	94.8	96.8	
	Yes	n	11	7	
		%	5.2	3.2	
Chronic kidney diseases (CKD)	No	n	210	214	0.015
		%	99.5	96.4	
	Yes	n	1	8	
		%	0.5	3.6	

## Discussion

COVID-19 displays a two-phase progression. The virus's direct impact causes the early phase, and an excess of cytokine release causes the late phase (21). As cholangiocytes (60%) in the liver contain the majority of the virus's receptor, angiotensin converting enzyme 2 (ACE2), SARS-CoV-2 can directly infect these cells (22). Due to the excessive inflammatory response in the liver, this condition generates an increase in proinflammatory cytokines, which furthers the disease's progression. Moreover, HS results in a rise in the inflammatory response and degeneration of the immune system in addition to the deterioration of liver functioning. This is the process that occurs with direct virus damage to the liver (2–11).

When HS is combined with insulin resistance and several other conditions, it is called metabolic syndrome. Metabolic syndrome is an endocrinopathy that starts with insulin resistance and is followed by systemic illnesses such as obesity, type 2 DM, dyslipidemia, hypertension, HS, and CAD. Insulin resistance causes more fat to accumulate in the liver, which leads to HS. The onset and severity of the disease may be related to several risk factors. It is claimed in the literature that control groups who were COVID-19-positive had a lower risk of HS than positive groups (7, 11, 23). Additionally, Palomar-Lever et al. and Corapli et al. discovered that CT-SS was greater in individuals with COVID-19 with HS. However, these studies only included patients who tested positive for the virus (2, 24).

A review research study found that, in addition to advanced age, comorbidities such as hypertension, DM, CAD, cerebrovascular disease, COPD, chronic hepatitis, and cancer greatly affect the severity and prognosis of COVID-19 (25). Another study found that 12% of the 1482 patients with COVID-19 hospitalized in the USA who were included in the study had a history of comorbidity. Of these 1,482 patients, 34.6% had chronic liver disease, 48.3% were obese, 49.7% had hypertension, 28.3% had DM, and 27.8% had CHD (26). The most prevalent comorbidities in patients who tested positive for COVID-19 are hypertension, CHD, and CAD, and they seem to be risk factors for severe consequences (27–29). The heart and vascular tissues contain high concentrations of ACE2, making them ideal locations for viral colonization. While DM was present in 21.5% of COVID-19 mortalities, it was found that it was present in 3.9% of survivors (30). The Chinese Center for Disease Control reported that when DM in the same group was taken into account, the death rate of more than 70,000 cases rose from 2.3% to 7.3% (20).

In a different study published in the literature, severe COVID-19 was most strongly correlated with immunosuppression, DM, and cancer; in addition, older age, male gender, hypertension and DM were linked to higher mortality. According to the additional clinical conditions of patients with COVID-19 with fatty liver, classifications were constructed in our study using LCA, an advanced statistical technique. No study on the classification of the disease group connected to the LCA was discovered when the literature was searched. Comparisons were made between the groups generated as a result of the classification in terms of survival times and death rates. According to the study's results, patients with HS who have DM, CAD, chronic rhythm disorders, COPD and CKD are more likely to die from COVID-19.

Patients with quantitatively recognizable HS may experience an overwhelming immune response to the SARS-CoV-2 virus, resulting in a cytokine storm that has a negative impact on the patients' prognosis. When using a chest CT on patients with COVID-19, it is crucial to assess the heart, liver and kidneys in addition to the lungs for additional disease. With knowledge of the patients' prognosis, close follow-up can be conducted in light of this information. As the intensity of the inflammation may worsen, some patients may not be suitable for home treatment. Therefore, when the patients are closely monitored by assessing this scenario, the recovery rate of the patients will increase since the condition of the patients with other disorders also affects the process.

The strength of our study is that we conducted a thorough investigation to assess the death and survival rates of individuals with HS who tested positive for COVID-19 while also taking into account the comorbidities. Furthermore, we believe that the amount of data and patient heterogeneity is adequate given the prevalence of COVID-19. The originality of the study is increased by applying LCA to evaluate the data. Such a study has not been documented in the literature based on these benefits. However, a limitation of our study is that not all disease categories and biochemical indicators could be evaluated in the study, and therefore a multicenter study involving different provincial hospitals could be conducted.

## Conclusion

The number of patients who died due to COVID-19 is rising daily as a result of the growing number of instances (1). By escalating the inflammatory response and degrading liver functioning, HS worsens the COVID-19 infection. HS, like other comorbidities, has a significant impact on how the disease develops. Moreover, patients may be suffering from various illnesses in addition to HS. The existence of these other disorders has a significant impact on how COVID-19 develops. In this study, an improved statistical method was used to explore the comorbidities of HS with COVID-19.

In patients with HS, the presence of DM, CAD, chronic rhythm disorders, COPD and CKD contributes to an increase in COVID-19 death rates. The high-risk group, which was generated as a result of the LCA, also had a significantly shorter survival time than the other group. Therefore, the comorbidities have a significant impact on the survival time and mortality risk in COVID-19-associated HS. Therefore, it is advised that the doctor should consider this information while deciding on the best course of action.

## Data availability statement

The raw data supporting the conclusions of this article will be made available by the authors, without undue reservation.

## Ethics statement

This retrospective and single-center study was approved by the Ethical Committee of Amasya University Sabuncuoglu Serefeddin Education and Research Hospital and was conducted according to the Declaration of Helsinki and Good Clinical Practice (10 December 2021, No: 2021/46711). The patients/participants provided their written informed consent to participate in this study.

## Author contributions

Conceived and designed the analysis and performed the analysis: OP. Collected data: AK. Contributed data or analysis tools: SC. Wrote the paper: OP, SC, and AK. All authors contributed to the article and approved the submitted version.

## Conflict of interest

The authors declare that the research was conducted in the absence of any commercial or financial relationships that could be construed as a potential conflict of interest.

## Publisher's note

All claims expressed in this article are solely those of the authors and do not necessarily represent those of their affiliated organizations, or those of the publisher, the editors and the reviewers. Any product that may be evaluated in this article, or claim that may be made by its manufacturer, is not guaranteed or endorsed by the publisher.

## References

[1] World Health Organization. COVID-19 Weekly Epidemiological Update 22. (2021). Available online at: https://www.who.int/docs/default-source/coronaviruse/situation-reports/weekly_epidemiological_update_22.pdf. (accessed December 2, 2021).

[2] Palomar-Lever A Barraza G Galicia-Alba J Echeverri-Bolaños M Escarria-Panesso R Padua-Barrios J. Hepatic steatosis as an independent risk factor for severe disease in patients with COVID-19: a computed tomography study. JGH Open. (2020) 4:1102–7. 32838045 10.1002/jgh3.12395 PMC7436487

[3] Targher G Mantovani A Byrne CD Wang XB Yan HD Sun QF. Detrimental effects of metabolic dysfunction-associated fatty liver disease and increased neutrophil-to-lymphocyte ratio on severity of COVID-19. Diabetes Metab. (2020) 46:505–7. 32505652 10.1016/j.diabet.2020.06.001 PMC7270805

[4] Yki-Järvinen H. Non-alcoholic fatty liver disease as a cause and a consequence of metabolic syndrome. Lancet Diabetes Endocrinol. (2014) 2:901–10. 24731669 10.1016/S2213-8587(14)70032-4

[5] Sanchis-Gomar F Lavie CJ Mehra MR Henry BM Lippi G. Obesity outcomes in COVID-19: when an epidemic and pandemic collide. Mayo Clin Proc. (2020) 95:1445–53. 32622449 10.1016/j.mayocp.2020.05.006 PMC7236707

[6] Lavie CJ Sanchis-Gomar F Henry BM Lippi G. COVID-19 and obesity: links and risks. Expert Rev Endocrinol Metab. (2020) 15:215–6. 10.1080/17446651.2020.1767589 32441223

[7] Medeiros AK Barbisan CC Cruz IR Araujo E.D. Libanio BB Albuquerque K.S. Higher frequency of hepatic steatosis at CT among COVID-19-positive patients. Abdom Radiol. (2020) 45:2748–54. 32683613 10.1007/s00261-020-02648-7 PMC7368629

[8] Ji D Qin E Xu J Zhang D Cheng G Wang Y. Non-alcoholic fatty liver diseases in patients with COVID-19: a retrospective study. J Hepatol. (2020) 73:451–3. 32278005 10.1016/j.jhep.2020.03.044 PMC7141624

[9] Nseir W Taha H Khateeb J Grosovski M Assy N. Fatty liver is associated with recurrent bacterial infections independent of metabolic syndrome. Dig Dis Sci. (2011) 56:328–34. 21562784 10.1007/s10620-011-1736-5

[10] Lefere S Tacke F. Macrophages in obesity and non-alcoholic fatty liver disease: Crosstalk with metabolism. JHEP Reports. (2019) 1:30–43. 32149275 10.1016/j.jhepr.2019.02.004 PMC7052781

[11] Gao F Zheng KI Wang X Yan H Sun QF Pan KH. Metabolic associated fatty liver disease increases coronavirus disease 2019 disease severity in nondiabetic patients. J Gastroenterol Hepatol. (2021) 36:204–7. 32436622 10.1111/jgh.15112 PMC7280625

[12] Hagenaars JA McCutcheon AL. Applied Latent Class Analysis. Cambridge University Press. (2002).

[13] Lanza ST Rhoades BL. Latent class analysis: an alternative perspective on subgroup analysis in prevention and treatment. Prev Sci. (2013) 14:157–68. 21318625 10.1007/s11121-011-0201-1 PMC3173585

[14] Weller BE Bowen NK Faubert S.J. Latent class analysis: a guide to best practice. J Black Psychol. (2020) 46:287–311.27492449

[15] Schneider KE Dayton L Rouhani S Latkin CA. Implications of attitudes and beliefs about COVID-19 vaccines for vaccination campaigns in the united states: a latent class analysis. Prevent Med Rep. (2021) 24:101584 34631397 10.1016/j.pmedr.2021.101584 PMC8493734

[16] Prokop M Van Everdingen W Van Rees Vellinga T Van Ufford HQ Stöger L Beenen L. CO-RADS: a categorical CT assessment scheme for patients suspected of having COVID-19-definition and evaluation. Radiology. (2020) 296:E97–104. 32339082 10.1148/radiol.2020201473 PMC7233402

[17] Zeb I Li D Nasir K Katz R Larijani VN Budoff MJ. Computed tomography scans in the evaluation of fatty liver disease in a population based study: the multi-ethnic study of atherosclerosis. Acad Radiol. (2012) 19:811–8. 22521729 10.1016/j.acra.2012.02.022 PMC3377794

[18] Piekarski J Goldberg HI Royal SA Axel L Moss AA. Difference between liver and spleen CT numbers in the normal adult: its usefulness in predicting the presence of diffuse liver disease. Radiology. (1980) 137:727–9. 6934563 10.1148/radiology.137.3.6934563

[19] Kawata R Sakata K Kunieda T Saji S Doi H Nozawa Y. Quantitative evaluation of fatty liver by computed tomography in rabbits. AJR Am J Roentgenol. (1984) 142:741–6. 6608234 10.2214/ajr.142.4.741

[20] Hill MA Mantzoros C Sowers JR. Commentary: COVID-19 in patients with diabetes. Metabolism. (2020) 107:154217. 32220611 10.1016/j.metabol.2020.154217 PMC7102643

[21] Huang C Wang Y Li X Ren L Zhao J Hu Y. Clinical features of patients infected with 2019 novel coronavirus in Wuhan, China. Lancet. (2020) 395:497–506. 31986264 10.1016/S0140-6736(20)30183-5 PMC7159299

[22] Hamming I Timens W Bulthuis MLC Lely AT Navis GJ van Goor H. Tissue distribution of ACE2 protein, the functional receptor for SARS coronavirus: A first step in understanding SARS pathogenesis. J Pathol A J Pathol Soc Gt Britain Irel. (2004) 203:631–7. 15141377 10.1002/path.1570 PMC7167720

[23] Tahtabasi M Hosbul T Karaman E Akin Y Kilicaslan N. Frequency of hepatic steatosis and its association with the pneumonia severity score on chest computed tomography in adult COVID-19 patients. World J Crit Care Med. (2021) 10:47–57. 34046310 10.5492/wjccm.v10.i3.47 PMC8131933

[24] Çorapli M Çil E Oktay C Kaçmaz H Çorapli G Bulut HT. Role of hepatosteatosis in the prognosis of COVID 19 disease. Clin Imaging. (2021) 80:1–5. 34214871 10.1016/j.clinimag.2021.06.034 PMC8234248

[25] Fang X Li S Yu H Wang P Zhang Y Chen Z. Epidemiological, comorbidity factors with severity and prognosis of COVID-19: a systematic review and meta-analysis. Aging (Albany NY). (2020) 12:12493. 32658868 10.18632/aging.103579 PMC7377860

[26] Garg S Kim L Whitaker M O'Halloran A Cummings C Holstein R. Hospitalization rates and characteristics of patients hospitalized with laboratory-confirmed coronavirus disease 2019—COVID-NET, 14 States, March 1–30, 2020. Morb Mortal Wkly Rep. (2020) 69:458. 10.15585/mmwr.mm6915e3 PMC7755063 32298251

[27] Yang J Zheng Y Gou X Pu K Chen Z Guo Q. Prevalence of comorbidities and its effects in patients infected with SARS-CoV-2: a systematic review and meta-analysis. Int J Infect Dis. (2020) 94:91–5. 32173574 10.1016/j.ijid.2020.03.017 PMC7194638

[28] Fang L Karakiulakis G Roth M. Are patients with hypertension and diabetes mellitus at increased risk for COVID-19 infection? Lancet Respir Med. (2020) 8:e21. 32171062 10.1016/S2213-2600(20)30116-8 PMC7118626

[29] Zhou F Yu T Du R Fan G Liu Y Liu Z. Clinical course and risk factors for mortality of adult inpatients with COVID-19 in Wuhan, China: a retrospective cohort study. Lancet. (2020) 395:1054–62. 32171076 10.1016/S0140-6736(20)30566-3 PMC7270627

[30] Yang JK Feng Y Yuan MY Yuan SY Fu HJ Wu BY. Plasma glucose levels and diabetes are independent predictors for mortality and morbidity in patients with SARS. Diabet Med. (2006) 23:623–8. 16759303 10.1111/j.1464-5491.2006.01861.x

